# Treatment with MOG-DNA vaccines induces CD4^+^CD25^+^FoxP3^+^ regulatory T cells and up-regulates genes with neuroprotective functions in experimental autoimmune encephalomyelitis

**DOI:** 10.1186/1742-2094-9-139

**Published:** 2012-06-22

**Authors:** Nicolás Fissolo, Carme Costa, Ramil N Nurtdinov, Marta F Bustamante, Victor Llombart, María J Mansilla, Carmen Espejo, Xavier Montalban, Manuel Comabella

**Affiliations:** 1 Centre d’Esclerosi Múltiple de Catalunya, CEM-Cat, Unitat de Neuroimmunologia Clínica, Hospital Universitari Vall d´Hebron (HUVH), Barcelona, Spain

**Keywords:** MS, EAE, DNA vaccines, Neuroprotection, Tolerance

## Abstract

**Background:**

DNA vaccines represent promising therapeutic strategies in autoimmune disorders such as multiple sclerosis (MS). However, the precise mechanisms by which DNA vaccines induce immune regulation remain largely unknown. Here, we aimed to expand previous knowledge existing on the mechanisms of action of DNA vaccines in the animal model of MS, experimental autoimmune encephalomyelitis (EAE), by treating EAE mice with a DNA vaccine encoding the myelin oligodendrocyte glycoprotein (MOG), and exploring the therapeutic effects on the disease-induced inflammatory and neurodegenerative changes.

**Methods:**

EAE was induced in C57BL6/J mice by immunization with MOG_35-55_ peptide. Mice were intramuscularly treated with a MOG-DNA vaccine or vehicle in prophylactic and therapeutic approaches. Histological studies were performed in central nervous system (CNS) tissue. Cytokine production and regulatory T cell (Treg) quantification were achieved by flow cytometry. Gene expression patterns were determined using microarrays, and the main findings were validated by real-time PCR.

**Results:**

MOG-DNA treatment reduced the clinical and histopathological signs of EAE when administered in both prophylactic and therapeutic settings. Suppression of clinical EAE was associated with dampening of antigen (Ag)-specific proinflammatory Th1 and Th17 immune responses and, interestingly, expansion of Treg in the periphery and upregulation in the CNS of genes encoding neurotrophic factors and proteins involved in remyelination.

**Conclusions:**

These results suggest for the first time that the beneficial effects of DNA vaccines in EAE are not limited to anti-inflammatory mechanisms, and DNA vaccines may also exert positive effects through hitherto unknown neuroprotective mechanisms.

## Background

The prevailing hypothesis to describe the pathogenesis of multiple sclerosis (MS) is that myelin destruction within the central nervous system (CNS) is due to antigen (Ag)-specific autoimmunity [1,2]. Although the majority of currently used drugs for MS treatment target immune responses, they are not selective for autoreactive T cells. Furthermore, while effective in some cases, current MS therapies may also alter host-protective immune responses. Ideally, treatment strategies in MS should aim to restore self-tolerance selectively to the pathogenic autoantigens while leaving the healthy immune system intact. One promising Ag-specific approach is DNA vaccination.

DNA vaccination involves the injection of naked DNA that encodes target proteins under the control of a eukaryotic promoter [3]. Although DNA immunization research has largely focused on eliciting protective immunity against a variety of infectious pathogens, the technology may prove to have important applications in autoimmune diseases such as MS [4,5]. Vaccination using naked DNA encoding self-Ag has been shown to protect and even reverse established disease in several autoimmune animal models for various diseases, including rheumatoid arthritis [6], insulin-dependent diabetes mellitus [7] and MS [8]. Notably, recent clinical trials in MS patients based on DNA vaccination with myelin basic protein (MBP) demonstrated that the therapy was safe and well tolerated, caused Ag-specific immune tolerance, and was associated with a reduction in MRI-measured disease activity [9,10]. Despite the positive results achieved by DNA vaccination in the treatment of autoimmune disorders, the precise mechanisms of action displayed by DNA vaccines remain undefined.

In the present study, we aimed to evaluate the precise mechanisms by which a DNA vaccine encoding the myelin oligodendrocyte glycoprotein (MOG) induces immune regulation and efficiently suppresses experimental autoimmune encephalomyelitis (EAE) in both prophylactic and therapeutic settings. We observed that DNA vaccination with MOG was proven to be effective in ameliorating disease severity and reversing clinically established EAE. Importantly, beneficial actions of DNA vaccines encoding MOG were associated with downregulation of Ag-specific Th1 and Th17 cellular immune responses, increased frequency of natural CD4^+^CD25^+^FoxP3^+^ regulatory T cells (Treg) and upregulation of neuroprotective genes.

## Methods

### Plasmid DNA

cDNAs of murine full-length MOG, MBP and proteolipid protein (PLP) were obtained by retrotranscription and PCR amplification of total RNA isolated from mouse brain using the following PCR primers: 5’-AAAGAATTCGATGGCCTGTTTGTGGAGCT-3’ and 5’-AAAGCGGCCGCCAGGAAGACACAACCATCACTCA-3’ for MOG; 5’-AAAGAATTCTAGCCTGGATGTGATGGCAT-3’ and 5’-AAAGCGGCCGCCAGGATTCGGGAAGGCTGA-3’ for MBP; 5’-AAAGAATTCAAGTGCCAAAGACATGGGCT-3’ and 5’-AAAGCGGCCGCGCTCAGAACTTGGTGCCT-3’ for PLP. Fragments containing the myelin proteins included EcoRI and NotI restriction sites that were used for cloning. PCR products were inserted into the pCi plasmid, a cytomegalovirus promoter driven mammalian expression vector (Promega, Mannheim, Germany), digested with EcoRI and NotI to create MOG-, MBP- and PLP-DNA, respectively. The empty vector (pCi) was used as control plasmid. Plasmid DNA was purified from the transformed E. coli strain DH5α and sequenced to verify integrity. Large-scale preparation of plasmid DNA was conducted using Qiagen plasmid kits (Qiagen, Hilden, Germany). Each preparation was checked by agarose gel electrophoresis, and DNA concentration was measured by optical density at 260 nm.

### Mice

Five- to 8-week-old female C57BL6/J mice were purchased from Harlan (Italy). Experiments were done according to the EU regulations and approved by our institutional Ethics Committee on Animal Experimentation.

### DNA vaccination

Mice were allocated to four groups and vaccinated with plasmid DNA vaccines encoding MOG, PLP, or MBP, or with the control plasmid (pCi). For prophylactic treatment, 100 μg DNA/mouse in phosphate-buffered saline (PBS) was injected intramuscularly in the tibialis anterior muscle at 28 and 14 days before EAE induction. For therapeutic treatment, mice received intramuscular (i.m.) injections of DNA (100 μg/mouse) at days 10 and 24 postimmunization (p.i.).

### EAE induction

Anesthetized mice were immunized by subcutaneous injections of PBS containing 50 μg of MOG_35-55_ (Proteomics Section, Universitat Pompeu Fabra, Barcelona, Spain) emulsified in complete Freund's adjuvant (Sigma Chemical, St Louis, MO, USA) supplemented with 2 mg/ml Mycobacterium tuberculosis H37RA (Difco Laboratories, Detroit, MI, USA). The animals received an additional intravenous injection of 150 ng Pertussis toxin in 100 μl PBS on the day of immunization and again 48 h later. Mice were weighted and examined daily for clinical signs of EAE, which were scored as follows: grade 0, no clinical disease; grade 1, tail weakness or tail paralysis; grade 2, hind leg paraparesis; grade 3, hind leg paralysis; grade 4, paraplegia with forelimb weakness or paralysis; grade 5, moribund state or death.

### Histopathology and immunohistochemistry

Animals were killed with carbon dioxide (>70%) at day 32 p.i. Brain and spinal cord were removed, fixed overnight in paraformaldehyde 4% and embedded in paraffin wax. Brain and spinal cord were cut into 4-μm-thick serial sections. For histopathology, sections were stained with hematoxylin and eosin (H&E), and Kluver-barrera (KB). For immunohistochemistry, endogenous peroxidase activity was blocked by incubating tissue sections in hydrogen peroxide (2%), methanol (70%) and PBS for 20 min. Ag unmasking was developed in citrate 10 mM (pH = 6), 10 mM Tris-EDTA (pH = 9), or protease type XIV (Sigma Chemical). Non-specific protein binding was blocked with 2% bovine albumin in PBS (blocking solution) at room temperature for 1 h. Sections were incubated overnight at 4 °C with the following primary antibodies diluted in blocking solution: rabbit anti-CD3 (DakoCytomation, Glostrup, Denmark) (T lymphocytes) dilution 1:100, Lycopersicon esculentum agglutinin (LEA) (Sigma Chemical) (macrophages/microglia) dilution 1:100, rabbit anti- glial fibrillary acidic protein (GFAP) (DakoCytomation) (astrocytes) dilution 1:500, rabbit anti-Olig2 (Millipore, Billerica, MA, USA) (oligodendrocytes) dilution 1:100, and mouse anti-200kD neurofilament heavy (SMI-32) (Abcam, Cambridge, UK) (axonal damage) dilution 1:100. All samples were incubated at room temperature for 1 h with the following secondary antibodies: biotinylated anti-rabbit or anti-mouse IgG (DAKO) (1:200 dilution in blocking solution); the avidin–biotin-peroxidase complex (ImmunoPure ABC Peroxidase Staining Kits, Pierce, IL USA) (1:100 dilution in PBS) was finally added for 1 h at room temperature. The peroxidase reaction was visualized with 2.5 mg/ml of 3,30-diaminobenzidine and 0.05% hydrogen peroxide. As a background control, the primary antibody (Ab) incubation was omitted. No signal was observed in any of the control slides.

Cell infiltration (H&E) was evaluated according to the following criteria: 0 - no lesion; 1 - cellular infiltration only in the meninges; 2, very discrete and superficial infiltrates in parenchyma; 3, moderate infiltrate (less than 25%) in the white matter; 4, severe infiltrates (less than 50%) in the white matter; 5, more severe infiltrates (more than 50%) in the white matter. Demyelination (KB staining) was scored as follows: 0 - no demyelination; 1, little demyelination, only around infiltrates and involving less than 25% of the white matter; 2, demyelination involving less than 50% of the white matter; 3, diffuse and widespread demyelination involving more than 50% of the white matter.

Three randomly chosen areas (1 mm^2^) along the spinal cord were analyzed in a blind manner. CD3-, GFAP- and LEA-positive cells were counted in infiltrates manually. SMI-32 and Olig2 quantification was performed with the ImageJ analysis software.

### Cytokine assays

Mice were killed 14 days p.i. with CO_2_ and spleens removed. Splenocytes were prepared by grinding the spleens through a wire mesh and cultured in 96-well plates at 2 × 10^5^ cells/well in a total volume of 200 μl of Iscoves modified Dulbecco's medium (PAA Laboratories GmbH, Pasching, Austria) supplemented with 10% HyClone® Fetal Clone I (Thermo Fisher Scientific, Waltham, MA, USA), 50 μmol/ml of 2-mercaptoethanol (Sigma Chemical), 2 mmol/ml of glutamine, 50 U/ml of penicillin and 50 mg/ml of streptomycin, all obtained from Gibco BRL (Paisley, UK). Cultures (three replicas) were stimulated with 10 μg/ml of MOG_35-55_. Cells cultured without any stimulus were used as baseline controls. Cultures were incubated in a humidified atmosphere at 5% CO_2_ and 37 °C for 72 h. After stimulation, supernatants were collected, and cytokine levels for IFN-γ, IL-10, IL-4, and IL-17 were determined with the FlowCytomix kit (Bender MedSystems, Burlingame, CA, USA) according to the manufacturer’s recommendations. GM-CSF (granulocyte-macrophage colony-stimulating factor) levels were measured using a commercially available ELISA (R&D, Minneapolis, MN, USA).

### Anti-MOG_35-55_ ab detection

Nunc-Immuno Plates (Nalgene Nunc International, Roskilde, Denmark) were coated overnight with 0.1 μg/well of MOG_35–55_. Serum samples were added in duplicate and after extensive washes were incubated with a secondary horseradish peroxidase-conjugated goat anti-mouse IgG (H + L). Upon addition of TMB Substrate Reagent Set (BD Pharmingen, San Jose, CA, USA), plates were read at 450 nm in an ELISA reader.

### Quantification of CD4^+^CD25^+^ FoxP3^+^ treg

Freshly isolated splenocytes (5 × 10^5^) were washed with PBS-azide and incubated with PerCP-labelled anti-CD45, APC-Cy7-labeled anti-CD4, and PE-Cy7-labeled anti-CD25 for 30 min at 4 °C. Cells were then washed, and the intracellular detection of FoxP3 was conducted using a commercial FoxP3 staining protocol (eBioscience, San Diego, CA, USA). Data acquisition was performed on a FACSCanto^TM^ and analyzed with the DIVA software (BD Pharmingen).

### Gene expression microarrays

Total mRNA was extracted using TRI reagent (Sigma Chemical) from CNS tissue. Expression was analyzed with the Affymetrix Mouse Gene 1.0 Array using the Ambion WT Expression kit (Applied Biosystems, Foster City, CA, USA) for target amplification and WT Terminal Labeling kit (Affymetrix, Santa Clara, CA, USA) for target labeling. The gene-level log-scaled robust multiarray analysis (RMA) was performed with the Affymetrix Expression Console software. Linear models for microarray data (LIMMA) R package [11] were used to identify differentially expressed genes between the MOG-DNA-treated and control plasmid-treated groups. Genes that were observed as differentially expressed by two-sample *t*-test with p-value <0.05 were considered significant. Gene Ontology (GO) term enrichment was performed by using Ontologizer 2.0 [12]. Data were analyzed with this program using a model-based gene set analysis (MGSA) [13]. This method significantly reduces the number of redundant categories returned by the classical gene-category analysis. Pathway enrichment analysis was carried out with Ingenuity Pathway Analysis (IPA). Microarray data are stored in the NCBI Gene Expression Omnibus repository and are available at http://www.ncbi.nlm.nih.gov/geo/ with the entry number GSE30482.

### Real-time quantitative RT-PCR

Total mRNA was extracted from CNS tissue and retrotranscribed as described above. RT-PCR was performed using the ABI-Applied Biosystems 7900 HT Thermal Cycler (Applied Biosystems) in 384 optical PCR plates. Each reaction contained 2 μl standard/cDNA template, 10 μl of TaqMan Universal PCR Master Mix, No AmpErase®, UNG, 1 μl of corresponding TaqMan® Gene expression assay (Applied Biosystems), and 7 μl of RNase free water following the standard PCR program suggested by the manufacturer. mRNA expression levels of glyceraldehyde-3-phosphate dehydrogenase *(Gapdh)*, brain-derived neurotrophic factor (*Bdnf)*, neurotrophin-5 (*Ntf5)*, platelet-derived growth factor alpha (*Pdgfa)FoxP3*, glial cell line-derived neurotrophic factor (GDNF) family receptor alpha like *(Gfral)*, GDNF family receptor alpha 2 (*Gfra2)*, GDNF family receptor alpha 4 (*Gfra4),* and semaphorin-3 F (*Sema3F)* were determined by RT-PCR relative quantification in MOG-DNA-treated and control plasmid-treated mice. Briefly, the threshold cycle (C_T_) value for each reaction, and the relative level of gene expression for each sample were calculated using the 2^-ΔΔCT^ method [14]. To correct for loading differences, the values were normalized according to the level of expression of the housekeeping gene, *Gapdh*, within each sample. Its C_T_ value was subtracted from that of the specific genes to obtain a ΔCT value. Differences (ΔΔCT) between the ΔCT values obtained for the control plasmid (calibrators) and the ΔCT values for the MOG-DNA groups were determined. The relative quantitative value was then expressed as 2^-ΔΔCT^, representing the fold change in gene expression normalized to the endogenous control and relative to the calibrators. Samples were determined in triplicates. Analysis was performed with the software SDS 2.3 (Applied Biosystems).

### Statistical analysis

Statistical analysis was performed by using the SPSS 17.0 package (SPSS Inc, Chicago, USA) for MS-Windows. Depending on the applicability conditions, Mann–Whitney test or Student’s *t*-test were used for comparisons of mean values between groups. Differences were considered statistically significant when p-values were below 0.05. Descriptive data are presented as mean values (standard error of the mean - SEM) unless otherwise stated.

## Results

### Vaccination with DNA encoding MOG protects against EAE induction

**Figure 1:**
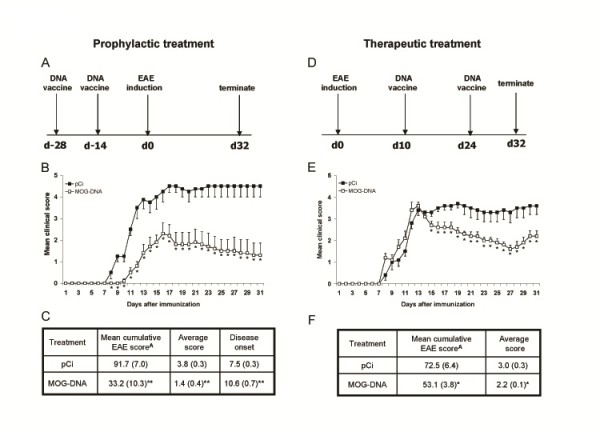
**Prophylactic and therapeutic treatment of EAE with MOG-DNA vaccination ameliorates the clinical signs of the disease.** EAE was induced in female C57BL6/J mice with MOG_35-55_ peptide in CFA. Mice were vaccinated with i.m. injections of 100 μg of DNA according to prophylactic (**A**) or therapeutic (**D**) protocols, as described in Methods. Two groups of mice (n = 5) were subjected to DNA treatment with the full-length MOG-DNA construct (□) or plasmid control (■) in prophylactic (**B**) or therapeutic (**E**) settings. Disease parameters in mice treated prophylactically and therapeutically are shown in tables (**C and F**). Mean clinical scores are plotted against the number of days after EAE induction. Disease scores are expressed as mean values (SEM). ^a^Indicates cumulative disease scores at 30 days p.i.; statistically significant differences obtained by Student’s t-tests are denoted with asterisks (*p < 0.05; **p < 0.005). Results shown are representative of two independent experiments.

To evaluate whether the injection of DNA encoding MOG cDNA can be effective in protecting mice from EAE induction, female C57BL6/J mice were first vaccinated with MOG-DNA or control plasmid (pCi) as described in Methods and depicted in Figure 1A. Mean clinical disease scores were significantly lower in mice vaccinated with the MOG-DNA plasmid vector compared with the control plasmid group (Figure 1B). Prophylactic MOG-DNA treatment also delayed disease onset [mean (SEM): 10.6 days (0.7) in MOG-DNA-treated mice vs. 7.5 days (0.3) in control plasmid-treated mice; *p* = 0.006], and reduced clinical severity [mean cumulative clinical scores: 1.4 (0.4) vs. 3.8 (0.3) in MOG-DNA-treated and control plasmid-treated mice respectively; *p* = 0.003] (Figure 1C).

In order to investigate whether these findings were specific for MOG-DNA vaccination or were also observed with other myelin autoantigens, mice were vaccinated with plasmid DNA vaccines encoding MBP or PLP (antigenic controls) using the same above-mentioned prophylactic protocol. As shown in Additional file 1, vaccination with DNA encoding MBP was also associated with a significant reduction in disease severity, although to a lesser degree compared with MOG-encoding DNA vaccines [mean cumulative clinical scores: 2.5 (0.1) vs. 3.8 (0.3) in MBP-treated and control plasmid-treated mice respectively; *p* = 0.003]. Finally, EAE disease course was similar between PLP-DNA-treated and control plasmid-treated mice (Additional file 1).

### MOG-DNA vaccination ameliorates ongoing EAE

We next addressed the question of whether MOG-DNA vaccination could reverse clinically established EAE. To this end, mice were left untreated until disease onset and subsequently vaccinated with MOG-DNA or vector DNA alone, as depicted in Figure 1D. Interestingly, therapeutic MOG-DNA treatment improved ongoing EAE, as reflected by the significant reduction in the mean cumulative EAE clinical score observed in MOG-DNA-treated mice compared with control plasmid-treated mice [2.2 (0.1) vs. 3.0 (0.3); *p* = 0.031] (Figure 1E and F).

These findings were specific for MOG-DNA treated mice, as mean EAE disease scores did not significantly differ between mice receiving therapeutic treatment with antigenic controls (MBP or PLP) and control plasmid (Additional file 1).

### CNS pathology is reduced by vaccination with MOG-encoding DNA

**Figure 2:**
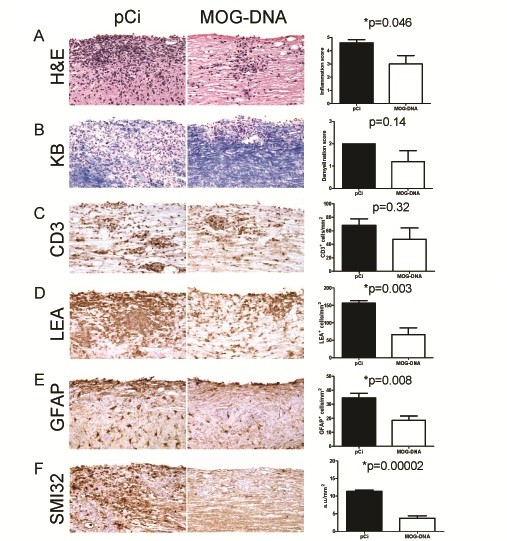
**MOG-DNA therapy decreased neuropathology associated with EAE.** Representative histopathologies of the spinal cord 32 days post-EAE induction in mice previously treated either with MOG-DNA or plasmid control according to a prophylactic protocol. Spinal cords were stained to evaluate cell infiltration (H&E; **A**), demyelination (KB; **B**), T cell infiltration (CD3; **C**), microglia/macrophage activation (LEA; **D**), reactive astrogliosis (GFAP; **E**), and axonal damage (SMI-32; **F**). Bar graphs represent mean number of cells/mm^2^ (SEM) observed in one representative experiment with five mice per group.

To assess whether EAE clinical improvement was accompanied by decreased neuropathology, histopathological studies were performed in CNS tissue from pCi and MOG-DNA treated mice. H&E and KB staining showed that mice treated with pCi had perivascular cuffs and extensive inflammatory infiltration in the white matter of the spinal cord (Figure 2A), as well as demyelination in areas with moderate to severe inflammatory infiltration (Figure 2B). Mice treated with MOG-DNA, on the other hand, were overall characterized by less severe CNS inflammation (Figure 2A) and significant reductions in microglia/macrophage activation (Figure 2D), astrogliosis (Figure 2E) and axonal damage (Figure 2F). Although demyelination and T cell infiltration were also reduced in MOG-DNA treated mice, differences did not reach statistical significance (Figures 2B and C respectively).

### MOG-DNA-treated animals show reduced MOG_35-55_-specific T cell responses

**Figure 3:**
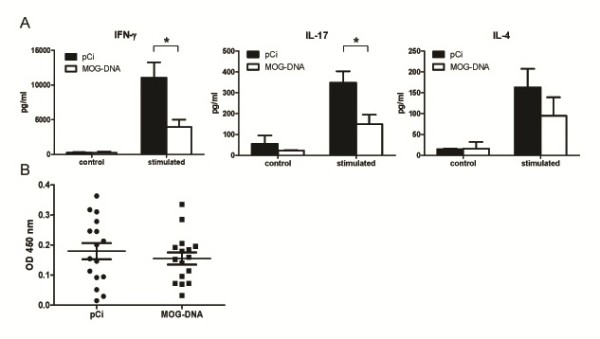
**Effect of MOG-DNA vaccination in T and B cell immune responses.** (**A**) Splenocytes isolated at day 14 p.i. from mice challenged for EAE induction with MOG_35-55_ and previously vaccinated with DNA (MOG or pCi) were incubated in the presence (stimulated) or absence (control) of MOG_35–55_ (10 μg/ml) for 48 h. Cytokine production in cell culture supernatants was determined by flow cytometry. Bars indicate mean values (SEM) in pg/ml of five mice per group. *p = 0.016 for IFN-γ and *p* = 0.013 for IL-17 (Student’s t-test). Results shown are representative of two independent experiments. (**B**) Serum samples from EAE mice treated with MOG-DNA or pCi were obtained at day 30 p.i., and anti-MOG_35-55_ IgG titers were determined by ELISA. Error bars indicate mean values and SEM. Data represent a pool of three different experiments.

Based on the findings of a reduction in both EAE disease course and CNS pathology in MOG-DNA treated mice, we performed additional experiments in order to characterize the mechanisms by which MOG-encoding DNA vaccines produce their beneficial effects in EAE. All these experiments were conducted in mice vaccinated according to the prophylactic protocol described in Methods. We first measured MOG_35-55_-induced T cell immune responses by determining ex vivo the levels of Th1, Th2, and Th17 cytokines using flow cytometry. As shown in Figure 3A, levels of the proinflammatory cytokines IFN-γ and IL17 were significantly reduced in mice vaccinated with MOG-encoding DNA compared to mice treated with control plasmid (*p* = 0.016 for IFN-γ; *p* = 0.013 for IL-17). However, levels of the anti-inflammatory cytokine IL-4 did not significantly differ between MOG-DNA treated and pCi-treated mice (*p* = 0.320; Figure 3A). Finally, levels of IL-10 in supernatants from stimulated mice splenocytes were below the detection limit of the technique.

Based on the IFN-γ and IL17 findings we also measured the levels of GM-CSF, which is known to play important roles in Th17 and Th1 cell function [15,16]. Supporting the IFN-γ and IL17 data, MOG-DNA treatment was associated with a trend towards decreased GM-CSF levels compared with plasmid-control mice [mean (SEM): 179.5 pg/ml (121.3) vs. 337.6 pg/ml (44.2) respectively; *p* = 0.065, Mann-Whitney *U* test)].

Altogether, these observations suggest that MOG-DNA vaccines exert some of their protective effects in EAE by inhibiting the secretion of proinflammatory cytokines such as IFN-γ and IL-17 rather than inducing Th2-type immune responses.

### Anti-MOG B cell responses are not altered by DNA vaccination

In order to characterize the autoreactive B-cell responses in vaccinated EAE mice, the presence of MOG-specific antibodies was determined in serum samples by ELISA. Anti-MOG antibodies were detected in all animals; however, Ab titers in MOG-DNA vaccinated mice were similar to those observed in control plasmid-treated mice (Figure 3B).

### MOG-DNA vaccination induces an expansion of Treg

**Figure 4:**
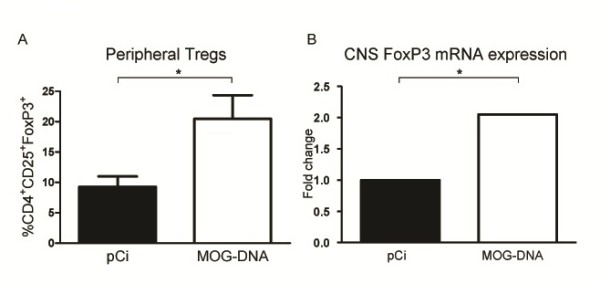
**Frequency of splenic Foxp3**^**+**^**-Treg in the periphery and mRNA expression of*Foxp3*in the CNS after MOG-DNA vaccination.** (**A**) Percentage of CD4^+^CD25^+^Foxp3^+^ Treg in the total CD4^+^ T cell population observed in spleens isolated from MOG- or pCi-treated mice (n = 6/group) at day 14 p.i. Frequency of Treg was determined by flow cytometry and bars represent mean percentage of positive cells (SEM). *p = 0.025 (Student’s t-test). (**B**) *Foxp3* mRNA expression levels in CNS tissue from DNA vaccinated animals at day 14 after EAE induction. *FoxP3* expression was determined by RT-PCR using *Gapdh* as endogenous control. Results are expressed as fold change in gene expression in MOG-DNA treated mice relative to plasmid control mice (calibrators). *p = 0.014 (Student’s t-test).

We next investigated whether treatment with MOG-DNA vaccines promoted the development of Treg in EAE animals. As shown in Figure 4A, the percentage of CD4^+^CD25^+^FoxP3^+^ Treg determined by flow cytometry was significantly higher in splenocytes from MOG-DNA-treated mice compared with animals treated with the empty plasmid [20.5% (3.9) vs. 9.3% (1.7), *p* = 0.025]. Given the potential of Treg to migrate into the CNS in response to inflammation, we also determined the expression of the Treg-specific transcription factor *FoxP3* in CNS tissue from EAE mice by RT-PCR. Interestingly, MOG-DNA vaccination was associated with a significant increase in CNS *FoxP3* mRNA expression levels compared with the control plasmid-treated condition (*p* = 0.014; Figure 4B). Taken together, these findings suggest that DNA vaccination with MOG may produce its beneficial effects through an expansion of the Treg population in the periphery and subsequent accumulation of Treg in the CNS.

### cDNA microarrays reveal downregulation of inflammatory genes and upregulation of neuroprotective genes in MOG-DNA-vaccinated mice

In order to characterize the mechanisms of action associated with the efficacy of MOG-DNA vaccines, we determined the gene expression profiles induced by DNA vaccination in the CNS using cDNA microarrays. A total of 2,462 genes were differentially expressed between MOG-DNA-treated and control plasmid-treated mice (*p* < 0.05). Of these, 1,382 genes were downregulated and 1,080 up-regulated in the MOG-DNA-treated group. In agreement with the pathological findings, gene enrichment analysis using the GO terms revealed the inflammatory response category to be the most represented among downregulated genes in mice vaccinated with MOG-DNA (Table 1). A detailed list of differentially expressed genes involved in the inflammatory process is provided in Additional file 2. In order to identify the cellular pathways induced by the effect of MOG-DNA vaccination, a more in depth functional enrichment analysis was performed with the Ingenuity software. Of note, one of the top enriched pathways that contained a high percentage of upregulated genes in the MOG-DNA-treated group was the axonal guidance signaling pathway (Additional file 3), which included genes coding for neurotrophic factors and proteins involved in the remyelination process. These findings pointed to a potential mechanism of action of MOG-DNA vaccines upregulating genes with neuroprotective roles. A search for additional neuroprotective genes (summarized in Table 2) was performed in order to validate microarray findings by an alternative technique.

**Table 1:** Top-scoring gene ontology categories down-regulated in mice vaccinated with MOG-encoding DNA

**GO ID**	**Category**	**MGSA**^**a**^	**Enrichment**^**b**^	**Expression**^**c**^
GO:0006954	Inflammatory response	0.935	4.1	Downregulated
GO:0002376	Immune system process	0.758	3.2	Downregulated
GO:0030133	Transport vesicle	0.719	3.9	Downregulated
GO:0000323	Lytic vacuole	0.609	4.3	Downregulated
GO:0015914	Phospholipid transport	0.511	4.1	Downregulated

GO-oriented analysis with differentially expressed genes obtained with microarrays from brain and spinal cord tissue was conducted in order to classify biologically related genes. Ontologizer 2.0 software was used for GO term enrichment analysis.

^a^MGSA: score obtained using a model-based gene set analysis. Only categories with a score >0.5 are represented.

^b^Enrichment values indicate how many times genes from the corresponding GO categories are overrepresented in the list of differentially expressed genes compared to the list of random genes.

^c^Expression: refers to the direction in gene expression observed in MOG-DNA treated mice (n = 5) versus plasmid control mice (n = 5). GO ID: GO identification.

**Table 2:** Summary of selected genes with neuroprotective functions found up-regulated with microarrays in MOG-DNA-treated mice

**Affymetrix probe set**	**Genes**	**Description**	**Gene ID**^**a**^	**FC**^**b**^	**I-value**
*Neurotrophic factors*					
10595050	*Gfral*	GDNF family receptor alpha like	404194	1.37	0.0186
10416340	*Gfra2*	GDNF family receptor alpha 2	14586	1.34	0.0030
10487787	*Gfra4*	GDNF family receptor alpha 4	14588	1.40	0.0475
10552938	*Ntf5*	Neurotrophin 5	78405	1.29	0.0212
10474399	*Bdnf*	Brain-derived neurotrophic factor	12064	1.38	0.0487
*Remyelination*					
10596747	*Sema3f*	Semaphorin 3 F	20350	1.32	0.0133
10526880	*Pdgfa*	Platelet-derived growth factor, alpha	18590	1.41	0.0389

Differentially expressed genes obtained with microarrays between MOG-DNA-treated mice (n = 5) and control plasmid-treated mice (n = 5) were estimated using Student’s *t* test, as described in Methods.

^a^Refers to Entrez gene ID.

^b^Fold change expression in MOG-DNA-treated mice versus control plasmid-treated mice. Data represent the mean ratio of five arrays. GDNF: glial cell line derived neurotrophic factor.

**Figure 5:**
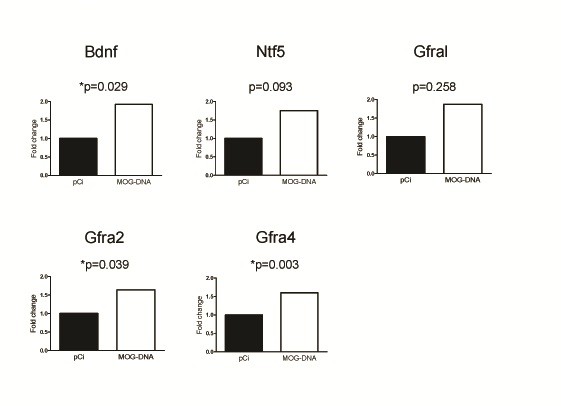
**Induction of neurotrophic factors in EAE mice treated with MOG-DNA.** mRNA expression levels for each gene were determined in CNS tissue by RT-PCR relative quantification, as described in Methods. *Gapdh* was used as endogenous control. Graphs indicate fold changes in gene expression in MOG-DNA treated mice relative to plasmid control mice (calibrators). ΔCT values were compared by means of Student’s t tests. Statistically significant differences are shown with asterisks.

As depicted in Figure 5, gene expression levels for neurotrophic factors determined by RT-PCR were overall found to be increased in mice vaccinated with MOG-encoding DNA compared with mice receiving control plasmid, and microarray differences were validated for *Bdnf* (*p* = 0.029); *Gfra2* (*p* = 0.039) and *Gfra4* (*p* = 0.003). A trend towards higher expression levels was also observed for *Ntf5* (*p* = 0.093), whereas differences for *Gfral* did not reach statistical significance (*p* = 0.258).

### Promotion of remyelination by MOG-DNA vaccination

**Figure 6:**
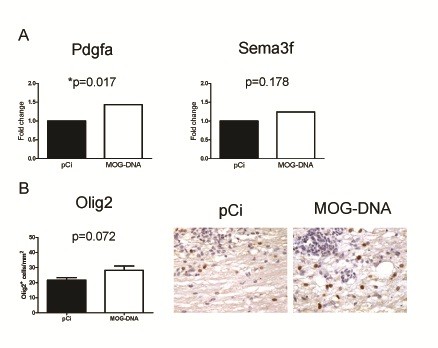
**Promotion of remyelination in mice vaccinated with MOG-DNA.** (**A**) Changes in mRNA expression levels for *Pdgfa* and *Sema3f* were determined in CNS from DNA-vaccinated mice on day 14 after EAE induction using RT-PCR relative quantification. A statistically significant increase was observed for *Pdgfa* (*p* = 0.017) in the group of animals vaccinated with MOG vs. pCi; for *Sema3f*, differences in expression did not reach statistical significance (*p* = 0.178). All values are normalized to *Gapdh* mRNA. (**B**) Expression of Olig2 by OPC was examined in spinal cords of MOG-DNA and plasmid control mice (brown staining). Quantification revealed that Olig2^+^-cells were increased in the CNS after treatment with DNA encoding MOG compared to control animals.

In microarray studies, two of the neuroprotective genes that were up-regulated in the MOG-DNA treated group, *Pdgfa* and *Sema3f*, are known to be involved in the remyelination process (Table 2) and hence were also selected for validation. As shown in Figure 6A, *Pdgfa* was found to be significantly overexpressed by RT-PCR in MOG-DNA vaccinated mice (*p* = 0.017), whereas the increased expression levels observed for *Sema3f* did not reach statistical significance (*p* = 0.178).

In order to provide histopathological evidence for a potential positive effect of DNA vaccines in remyelination, we also determined the expression of the transcription factor Olig2, a marker strongly expressed in oligodendrocyte progenitor cells (OPC) [17], in CNS tissue of vaccinated mice. Interestingly, a trend towards higher numbers of Olig2^+^ progenitor cells was observed in MOG-DNA-treated mice compared with plasmid control mice (*p* = 0.07; Figure 6B), suggesting that OPC are augmented in demyelinated CNS lesions from mice vaccinated with MOG-DNA.

Altogether these findings point to a potential neuroprotective effect of MOG-DNA vaccines favouring the remyelination process.

## Discussion

In this study, we investigated on the mechanisms involved in the therapeutic effects of vaccination with plasmid DNA encoding the encephalitogenic MOG protein on the inflammatory and neurodegenerative processes taking place during EAE disease course. We observed that vaccination with MOG-DNA improved both, prophylactically and therapeutically, clinical and histopathological signs of EAE. Mechanistic studies showed that protection from disease by MOG-DNA was accompanied by (*i*) decreased levels of the pro-inflammatory cytokines IFN-γ and IL-17; (ii) expansion of Treg in the periphery and increased mRNA expression levels in the CNS of the Treg-specific transcription factor FoxP3; (iii) down-regulation of genes involved in the inflammatory process and up-regulation of genes with neuroprotective functions.

A DNA vaccine encoding the full-length MBP molecule (BHT-3009) has been used in clinical trials to evaluate safety in MS patients [9,10]. DNA vaccines were found to be safe and demonstrated efficacy reducing brain MRI lesion activity. Although previous studies have mostly been performed on MBP, in our study we focused on MOG as the primary target autoantigen. Even though it is only a minor protein constituent of myelin (less than 0.05%), MOG is exclusively expressed in the CNS and is located on the surface of the myelin sheaths that surround neuronal axons [18]. Besides, in several animal models immunization with MOG causes inflammatory demyelinating lesions that are virtually indistinguishable from the active demyelinating plaques characteristic of MS [19].

Prophylactic treatment of EAE mice with MOG-DNA vaccines decreased disease severity, delayed disease onset, and reduced inflammation- and axonal damage-related CNS pathology. Unexpectedly, one of the antigenic controls, MBP, had also a positive effect on EAE disease course, albeit it did not modify the onset of disease. Of note, therapeutic treatment with plasmids encoding MOG, but not with MBP or PLP, resulted in amelioration of ongoing EAE. These data may set the rationale for the use of MOG-based DNA vaccines to treat MS patients, rationale that is further reinforced by the positive results observed in the mechanistic experiments conducted in the study and discussed below.

The profile of T cell reactivity to MOG_35-55_ after MOG-DNA vaccination showed a dramatic impairment of the IFN-γ and IL-17 responses at the peak of the disease. This may explain why DNA vaccination suppresses EAE, since these cytokines are known to be crucial in EAE [20,21]. The suppressive effect of DNA vaccination was both Th1 and Th17 cell-specific, as only IFN-γ and IL-17 responses were affected in the spleen, and the expression of relevant Th2 cytokines such as IL-4 and IL-10 were not altered by DNA vaccination. Although a beneficial Th2 bias has been reported in EAE [22,23], in our study we could not detect any measurable alteration of the Th1/Th2 balance of encephalitogenic T cells. In this regard, such Th2-driven effect was only observed when the myelin Ag present in the DNA vaccine were delivered along with DNA encoding IL-4 [22].

Comparable anti-MOG Ab titers were obtained in the blood of DNA-treated and control animals. This finding is most likely explained by the higher induction of anti-MOG antibodies in animals exhibiting clear clinical signs of EAE (control group) and the increase in the levels of anti-MOG antibodies induced by MOG-encoding DNA vaccines (MOG-DNA group) [24].

An interesting finding in our study was the expansion of Treg in the periphery observed in mice treated with MOG-DNA vaccines, together with an increase of mRNA FoxP3 expression in the CNS. Unfortunately, the Treg suppressive function and proportion of CNS infiltrating Treg cells was not investigated in our study and should be considered in future studies of DNA treatment in EAE. Absence of CD4^+^CD25^+^FoxP3^+^ natural suppressor T cells has been shown to enhance the development of T cell-mediated autoimmunity, whereas adoptive transfer of these cells was associated with opposite effects [25,26]. Considering that Th17 and induced Treg are activated through reciprocal mechanisms [27,28], the reduced IL-17 production by Th17 cells reported herein could be caused by enhanced activation of induced Treg during DNA vaccination. Contrary to our data, treatment of rat MOG_91-108_-EAE with a plasmid DNA vaccine encoding the MOG_91-108_ peptide was found to reduce the expression of FoxP3 in the periphery [29] or to have no effect on CD25 expression [30]. Our results are, however, in line with previous reports showing that DNA vaccination is able to induce Treg and protect animals against other organ specific autoimmune diseases such as autoimmune diabetes [31] and uveitis [32]. Considering the reports on impaired number and function of Treg in MS [33-35], therapies associated with an expansion of this suppressor T cell population may be desirable to treat patients with MS.

Another interesting finding in the present study was the up-regulation of genes encoding neurotrophic factors and proteins involved in remyelination in the CNS of MOG-DNA treated mice. In this context, while a variety of anti-inflammatory mechanisms have been described for DNA treatment in EAE (i.e., down-regulation of myelin protein-specific Th1 immune responses [36], increased Th2 responses [22], induction of IL-10 producing type 1 regulatory T cells [37] and up-regulation of IFNβ [30]), potential neuroprotective effects of DNA vaccines were not previously reported and represent a novel finding.

A number of studies support a role of neurotrophic factors such as the neurotrophins BDNF and Ntf5, and the growth factor GDNF together with its receptors (GFRAL, GFRA1-4) in neuroprotective and anti-inflammatory activities in both EAE and MS [38-42]. Particularly, numerous studies report positive effects of BDNF directly in the CNS stimulating tissue repair after traumatic injury [43]. The neuroprotective activities performed by neurotrophic factors also include improvement of myelin repair by stimulating the proliferation of OPC or by enhancing oligodendrocyte regeneration [38]. In the current study, MOG-DNA vaccines enhanced the expression in CNS tissue of genes involved in neuroprotection such as *BdnfNtf5*, and *Gdnf* receptors. These findings may represent one of the mechanisms of action whereby MOG-DNA vaccination exerts its protective effects in EAE restoring the injured CNS tissue via an increased production of neurotrophic factors.

Related with the potential induction of neuroprotective effects shown by MOG-DNA vaccines, an up-regulation of genes involved in remyelination such as *Pdgfa*[44] was also observed in treated mice. During development, OPC numbers are limited by the supply of Pdgfa and, for instance, previous studies have suggested the possibility of increasing the OPC population density in demyelinating areas by artificially enhancing Pdgfa supply [45]. Moreover, experiments in transgenic mice for the human PDGFA revealed increased oligodendrocyte generation and survival that promoted remyelination of chronic lesions [46]. In this setting, therapies that boost PDGFA production in the CNS tissue may have an attractive added value in MS. Although we do not have a direct evidence of remyelination, in our study the increased expression of Olig2+ cells induced by MOG-DNA treatment indicates augmented numbers of OPC in the spinal cords of the animals. Ultimately, these cells may have the potential to migrate, proliferate and, subsequently, differentiate into myelin-forming cells.

Altogether, these results suggest that the protective effects of MOG-DNA vaccines in EAE are not limited to anti-inflammatory mechanisms, and DNA vaccines may also act inducing neuroprotection. These findings have important clinical implications. In a complex disorder like MS in which two distinct components, inflammatory and neurodegenerative, clearly contribute to disease phenotype, therapeutic strategies targeting both components should prove to be more beneficial for the disease than those targeting each component in isolation. It is important to highlight that the neurodegenerative component of the disease underlies the treatment-resistant progressive forms of MS which are associated with important neurological disability. In this setting, therapies with neuroprotective effects are needed for the disease.

## Conclusions

In the present study DNA vaccination with MOG has demonstrated efficacy in improving EAE disease severity when administered both prophylactically and therapeutically to mice. Furthermore, the beneficial effects of MOG-DNA vaccines were accompanied by a reduction of pro-inflammatory cytokines, expansion of Treg, and up-regulation of neuroprotective genes. Although these positive findings are based on the animal model of MS, altogether our data may provide the rationale for using MOG-DNA vaccines to treat MS patients in phase I/II clinical trials.

## Abbreviations

Ag, Antigen; Ab, Antibody; BDNF, Brain-derived neurotrophic factor; CNS, Central nervous system; EAE, Experimental autoimmune encephalomyelitis; GDNF, Glial cell line-derived neurotrophic factor; GFAP, Glial fibrillary acidic protein; GO, Gene ontology; H&E, Hematoxylin and eosin; KB, Kluver-barrera; LEA, Lycopersicon esculentum agglutinin; MBP, Myelin basic protein; MOG, Myelin oligodendrocyte glycoprotein; MS, Multiple sclerosis; OPC, Oligodendrocyte progenitor cells; PDGFA, Platelet-derived growth factor alpha; PBS, Phosphate buffered saline; PLP, Proteolipid protein; Treg, Regulatory T cells.

## Competing interests

The authors declare that they have no competing interests.

## Authors’ contributions

N.F. designed, performed, analyzed and interpreted all the experiments and wrote the manuscript. C.C planned and performed experiments. R.N. interpreted the microarray experiments. M.B. contributed to the experimental design, V.L. and M.M. provided technical assistance. C.E. contributed to discussion. X.M. supervised the study. M.C. contributed to the experimental design, analyzed and interpreted all acquired data and helped to write the manuscript. All authors read and approved the final manuscript.

## Supplementary Material

Additional file 1 ** Figure S1 Prophylactic and therapeutic DNA treatment of EAE with PLP and MBP antigenic controls.** EAE was induced in C57BL6/J mice with MOG_35-55_ peptide in CFA. (a) Mice were treated in prophylactic (a and b) or therapeutic settings (c and d), as previously described in Methods. Five mice in each group were vaccinated with DNA containing the full-length MBP-DNA construct (○), PLP-DNA construct (Δ), or plasmid control (■). Mean clinical scores are plotted against the number of days after EAE induction. Disease scores are expressed as mean values (SEM). ^a^Indicates cumulative disease scores on 30 days p.i. Statistically significant differences obtained by Student’s-t tests are denoted with asterisks (*p < 0.05). Click here for file

Additional file 2 ** Table S1 Genes involved in the inflammatory process that are down-regulated by MOG-DNA treatment.** Differentially expressed genes obtained with microarrays between MOG-DNA-treated mice (n = 5) and control plasmid-treated mice (n = 5). Click here for file

Additional file 3 ** Table S2 Top enriched pathways identified among differentially expressed genes.** Pathways ranked by percentage of up-regulated genes in mice vaccinated with MOG-DNA. Click here for file
