# Supplementary material for: Treatment with MOG-DNA vaccines induces CD4+CD25+FoxP3+ regulatory T cells and up-regulates genes with neuroprotective functions in experimental autoimmune encephalomyelitis
Source: J Neuroinflammation. 2012 Jun 22;9:139. doi: 10.1186/1742-2094-9-139 (PMC3464883; doi:10.1186/1742-2094-9-139)
Supplement: Additional file 1 — Figure S1 Prophylactic and therapeutic DNA treatment of EAE with PLP and MBP antigenic controls. EAE was induced in C57BL6/J mice with MOG35-55 peptide in CFA. (a) Mice were treated in prophylactic (a and b) or therapeutic settings (c and d), as previously described in Methods. Five mice in each group were vaccinated with DNA containing the full-length MBP-DNA construct (○), PLP-DNA construct (Δ), or plasmid control (■). Mean clinical scores are plotted against the number of days after EAE induction. Disease scores are expressed as mean values (SEM). aIndicates cumulative disease scores on 30 days p.i. Statistically significant differences obtained by Student’s-t tests are denoted with asterisks (*p < 0.05). [file 1742-2094-9-139-S1.doc]

**Additional File 1: Figure S1**
