# Supplementary material for: Treatment with MOG-DNA vaccines induces CD4+CD25+FoxP3+ regulatory T cells and up-regulates genes with neuroprotective functions in experimental autoimmune encephalomyelitis
Source: J Neuroinflammation. 2012 Jun 22;9:139. doi: 10.1186/1742-2094-9-139 (PMC3464883; doi:10.1186/1742-2094-9-139)
Supplement: Additional file 2 — Table S1 Genes involved in the inflammatory process that are down-regulated by MOG-DNA treatment. Differentially expressed genes obtained with microarrays between MOG-DNA-treated mice (n = 5) and control plasmid-treated mice (n = 5). [file 1742-2094-9-139-S2.doc]

**Additional File 2: Table S1.** Genes involved in the inflammatory process that are down-regulated by MOG-DNA treatment

| **Affymetrix probe set** | **Gene name** | **Description** | **Gene ID^a)^** | **FC^b)^** | **p-value** |
| --- | --- | --- | --- | --- | --- |
| *Cytokines and cytokine receptors* | | | | | |
| 10450501 | *Tnf* | tumor necrosis factor | 21926 | 1.61 | 0.0009 |
| 10492540 | *Il12a* | interleukin 12a | 16159 | 1.26 | 0.0050 |
| 10520452 | *Il6* | interleukin 6 | 16193 | 1.25 | 0.0066 |
| 10541246 | *Il17ra* | interleukin 17 receptor A | 16172 | 1.42 | 0.0084 |
| 10366586 | *Ifng* | interferon gamma | 15978 | 1.44 | 0.0104 |
| 10545135 | *Il12rb2* | interleukin 12 receptor, beta 2 | 16162 | 1.33 | 0.0125 |
| 10557342 | *Il21r* | interleukin 21 receptor | 60504 | 1.31 | 0.0249 |
| 10487588 | *Il1a* | interleukin 1 alpha | 16175 | 2.04 | 0.0262 |
| *Chemokines and adhesion molecules* | | | | | |
| 10389064 | *Ccl1* | chemokine (C-C motif) ligand 1 | 20290 | 1.97 | 0.0002 |
| 10583519 | *Icam1* | intercellular adhesion molecule 1 | 15894 | 1.52 | 0.0010 |
| 10485405 | *Cd44* | CD44 antigen | 12505 | 1.51 | 0.0022 |
| 10531420 | *Cxcl11* | chemokine (C-X-C motif) ligand 11 | 56066 | 1.29 | 0.0029 |
| 10532711 | *Cmklr1* | chemokine-like receptor 1 | 14747 | 1.74 | 0.0031 |
| 10523156 | *Cxcl2* | chemokine (C-X-C motif) ligand 2 | 20310 | 1.83 | 0.0031 |
| 10501608 | *Vcam1* | vascular cell adhesion molecule 1 | 22329 | 1.29 | 0.0034 |
| 10433114 | *Itga5* | integrin alpha 5 (fibronectin receptor alpha) | 16402 | 1.75 | 0.0035 |
| 10379524 | *Ccl11* | chemokine (C-C motif) ligand 11 | 20292 | 1.33 | 0.0039 |
| 10557591 | *Itgal* | integrin alpha L | 16408 | 1.72 | 0.0041 |
| 10364262 | *Itgb2* | integrin beta 2 | 16414 | 1.83 | 0.0053 |
| 10557862 | *Itgam* | integrin alpha M | 16409 | 1.66 | 0.0059 |
| 10432957 | *Itgb7* | integrin beta 7 | 16421 | 1.52 | 0.0065 |
| 10590635 | *Ccr5* | chemokine (C-C motif) receptor 5 | 12774 | 1.66 | 0.0098 |
| 10379511 | *Ccl2* | chemokine (C-C motif) ligand 2 | 20296 | 2.04 | 0.0146 |
| 10473125 | *Itga4* | integrin alpha 4 | 16401 | 1.52 | 0.0159 |
| 10598004 | *Ccr1* | chemokine (C-C motif) receptor 1 | 12768 | 2.06 | 0.0194 |
| 10531415 | *Cxcl10* | chemokine (C-X-C motif) ligand 10 | 15945 | 1.48 | 0.0366 |
| *Transcription factors* | | | | | |
| 10391301 | *Stat3* | signal transducer and activator of transcription 3 | 20848 | 1.35 | 0.0021 |
| 10366956 | *Stat6* | signal transducer and activator of transcription 6 | 20852 | 1.49 | 0.0022 |
| 10376060 | *Irf1* | interferon regulatory factor 1 | 16362 | 1.80 | 0.0095 |
| 10367224 | *Stat2* | signal transducer and activator of transcription 2 | 20847 | 1.30 | 0.0123 |
| 10346191 | *Stat1* | signal transducer and activator of transcription 1 | 20846 | 1.50 | 0.0148 |
| 10390328 | *Tbx21* | T-box transcription factor TBX21 | 57765 | 1.24 | 0.0161 |
| *Demyelination and axonal injury* | | | | | |
| 10481056 | *Notch1* | Notch gene homolog 1 | 18128 | 1.30 | 0.0029 |
| 10465314 | *Capn1* | calpain 1 | 12333 | 1.34 | 0.0029 |
| 10379228 | *Nos2* | nitric oxide synthase 2, inducible | 18126 | 2.43 | 0.0037 |
| 10450508 | *Lta* | lymphotoxin A | 16992 | 1.21 | 0.0042 |
| 10491699 | *Fgf2* | fibroblast growth factor 2 | 14173 | 1.36 | 0.0093 |

Differentially expressed genes obtained with microarrays between MOG-DNA-treated mice (n=5) and control plasmid-treated mice (n=5) were estimated using the Student’s t test, as described in Methods.

^a)^Refers to Entrez gene ID.

^b)^Fold change expression in MOG-DNA-treated versus control plasmid-treated mice.
