# Supplementary material for: Treatment with MOG-DNA vaccines induces CD4+CD25+FoxP3+ regulatory T cells and up-regulates genes with neuroprotective functions in experimental autoimmune encephalomyelitis
Source: J Neuroinflammation. 2012 Jun 22;9:139. doi: 10.1186/1742-2094-9-139 (PMC3464883; doi:10.1186/1742-2094-9-139)
Supplement: Additional file 3 — Table S2 Top enriched pathways identified among differentially expressed genes. Pathways ranked by percentage of up-regulated genes in mice vaccinated with MOG-DNA. [file 1742-2094-9-139-S3.doc]

**Additional file 3: Table S2.** Top enriched pathways identified among differentially expressed genes

| **Pathway** | **p-value** | **up-regulated genes^a)^** | **down-regulated genes^b)^** | **%^c^** |
| --- | --- | --- | --- | --- |
| G-Protein coupled receptor signalling | 0.0001 | 35 | 44 | 44 |
| Axonal guidance signalling | 0.003 | 26 | 32 | 42 |
| NRF2-mediated oxidative stress response | 0.003 | 9 | 20 | 31 |
| Endothelin-1 signalling | 0.00003 | 8 | 27 | 23 |
| Leukocyte extravasation signalling | 0.00001 | 5 | 37 | 12 |
| Glucocorticoid receptor signalling | 0.0006 | 5 | 39 | 11 |
| NF-κB signalling | 0.000001 | 4 | 36 | 10 |

A total of 111 enriched pathways were defined by applying 0.01 as an enrichment p-value threshold, which contained 513 differentially expressed genes. Top seven pathways contain 266 (52%) of all differentially expressed genes and have very good enrichment significance (p<0.004). Pathways are ranked by percentage of up-regulated genes in mice vaccinated with MOG-DNA.

^a)^Number of genes up-regulated by MOG-DNA treatment.

^b)^Number of genes down-regulated by MOG-DNA treatment.

^c)^Percentage of genes up-regulated by MOG-DNA vaccines.
